# Supplementary figures and images for: Mutation Y453F in the spike protein of SARS-CoV-2 enhances interaction with the mink ACE2 receptor for host adaption
Source: PLoS Pathog. 2021 Nov 8;17(11):e1010053. doi: 10.1371/journal.ppat.1010053 (PMC8601601; doi:10.1371/journal.ppat.1010053)

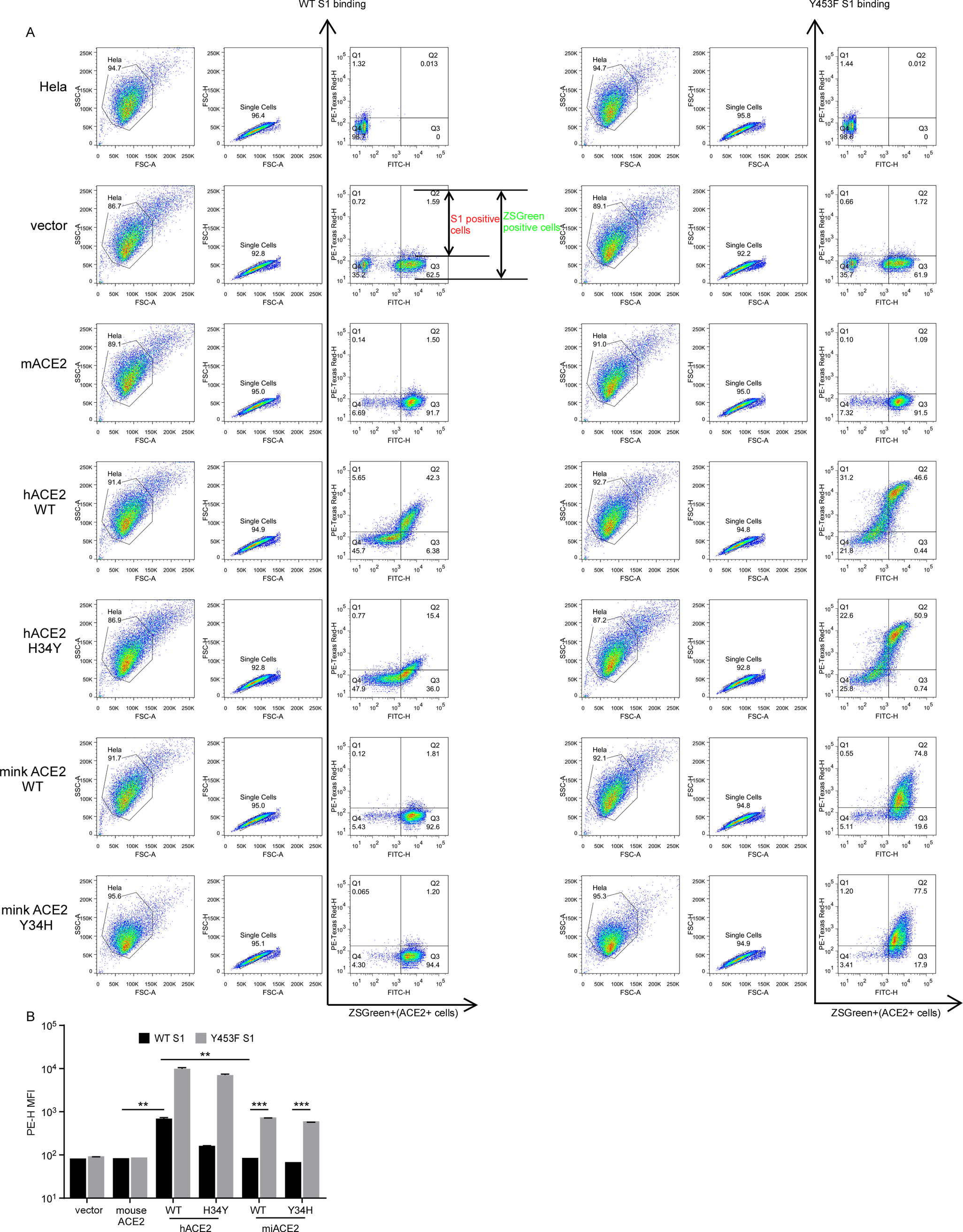

Supplement: S1 Fig — (A) The main cell population was identified and gated on Forward and Side Scatter. Single cells were further gated on FSC-A and FSC-H. The gated cells were plotted by FITC-H (zsGreen, the ACE2 -expressing population) and PE-Texas Red-H (S1-Fc bound population). The PE-Texas Red-H positive cell population was plotted as a histogram to show the S1-Fc positive population as in Fig 2A. The binding efficiency was defined as the percent of S1-Fc binding cells among the zsGreen-positive cells. (B) The MFI values of ACE2+ cells incubated with S1 proteins as indicated were plotted. Shown are FACS plots representative of those used for the calculations of binding efficiencies of ACE2 orthologs with S1-Fc. All binding assays were performed in duplicate. (TIF) [file ppat.1010053.s001.tif]

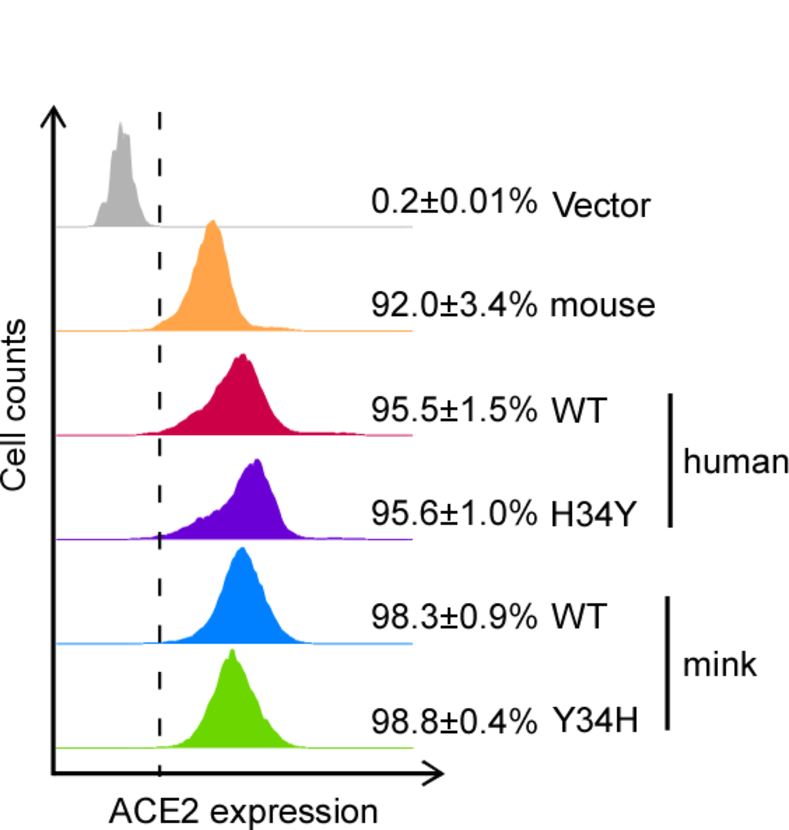

Supplement: S2 Fig — HeLa cells transduced with lentiviruses (pLVX-IRES-zsGreen) expressing mouse, human (WT or H34Y) or mink ACE2 (WT or Y34H) were incubated with rabbit polyclonal antibody (Sino Biological Inc. China, Cat: 10108-T24) against ACE2. The cells were washed and then stained with 2μg/mL goat anti-rabbit IgG (H+L) conjugated with Alexa Fluor 568 for flow cytometry analysis. The cell surface ACE2 was calculated as the percent of Alex Fluor 568-positive cells among the zsGreen-positive cells. This experiment was repeated twice with similar results. (TIF) [file ppat.1010053.s002.tif]

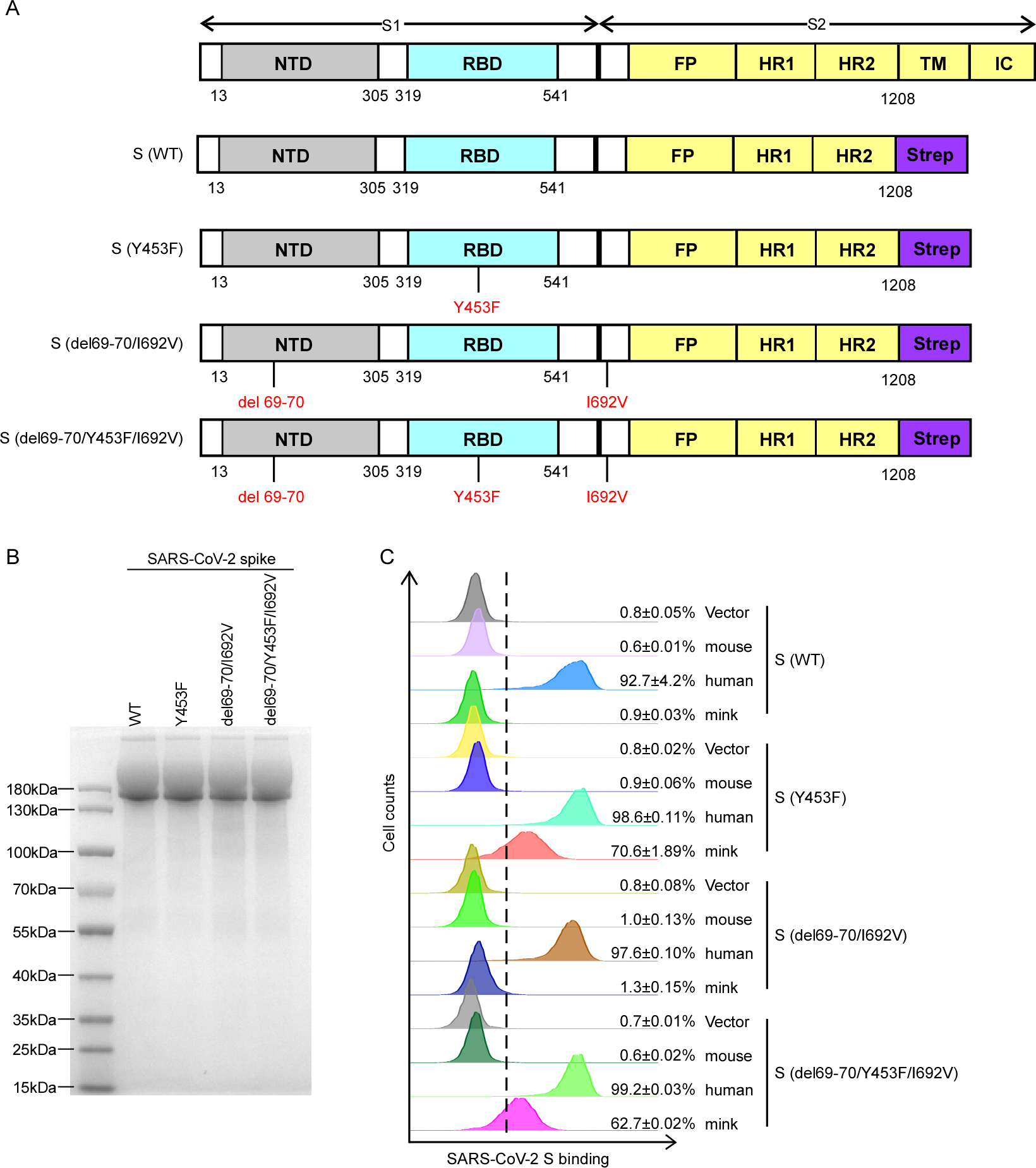

Supplement: S3 Fig — (A) Scheme of the WT and mutant spike proteins. (B) The purified proteins were analyzed by SDS-PAGE with Coomassie blue staining. (C) HeLa cells were transduced with human, mouse or mink ACE2 as indicated. The transduced cells were incubated with the WT or mutants spike protein of SARS-CoV-2 C-terminally fused with a His tag and then stained anti-His-PE for flow cytometry analysis. Values are expressed as the percent of cells positive for S-Fc among the ACE2-expressing cells (zsGreen1+ cells) and shown as the means ± SD from 3 biological replicates. These experiments were independently performed three times with similar results. (TIF) [file ppat.1010053.s003.tif]

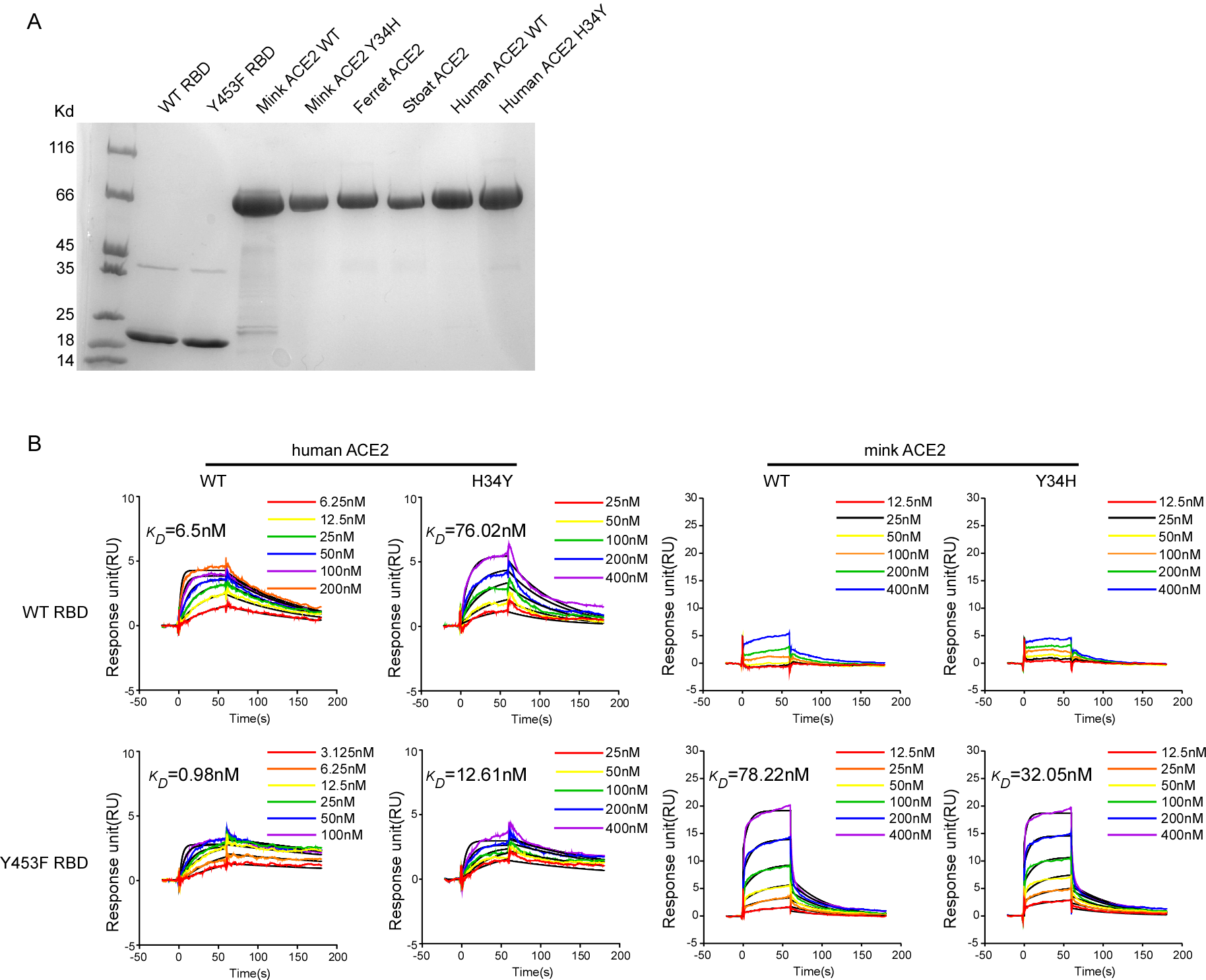

Supplement: S4 Fig — (A) The N-terminal peptidase domain of each ACE2 variant (residues Met1-Asp615), WT or Y453F SARS-CoV-2 RBD (residues Thr333-Pro527) were expressed and purified as described in the Materials and Methods. The purified proteins were analyzed by SDS-PAGE with Coomassie blue staining. (B) The binding kinetics of ACE2 variant proteins (human or mink) with recombinant WT or Y453F SARS-CoV-2 RBD were obtained using the BIAcore. ACE2 proteins were captured on the chip, and serial dilutions of RBD were then injected over the chip surface. Experiments were performed three times with similar results, and one set of representative data is displayed. (TIF) [file ppat.1010053.s004.tif]

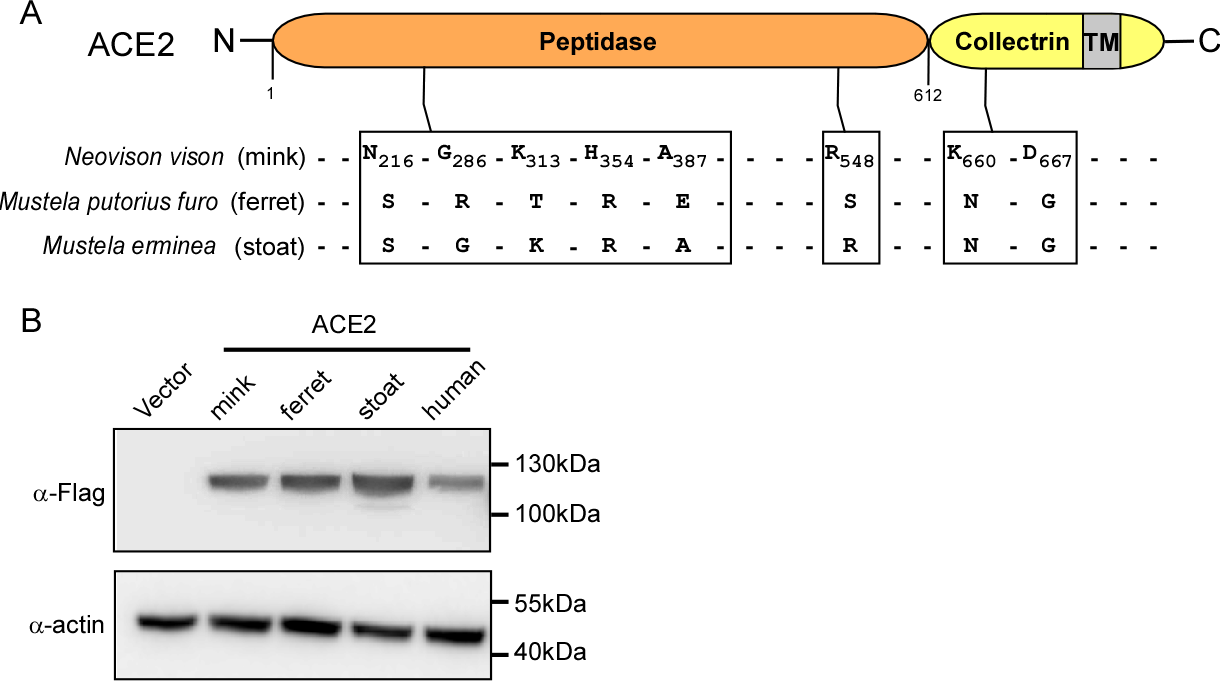

Supplement: S5 Fig — (A) Alignment of ACE2 orthologs of the Mustelidae species mink, ferret and stoat. (B) Representative immunoblots of HeLa cells transduced with lentiviruses expressing FLAG-tagged ACE2 orthologs as indicated. Actin used as the loading control. These experiments were independently performed twice with similar results. (TIF) [file ppat.1010053.s005.tif]

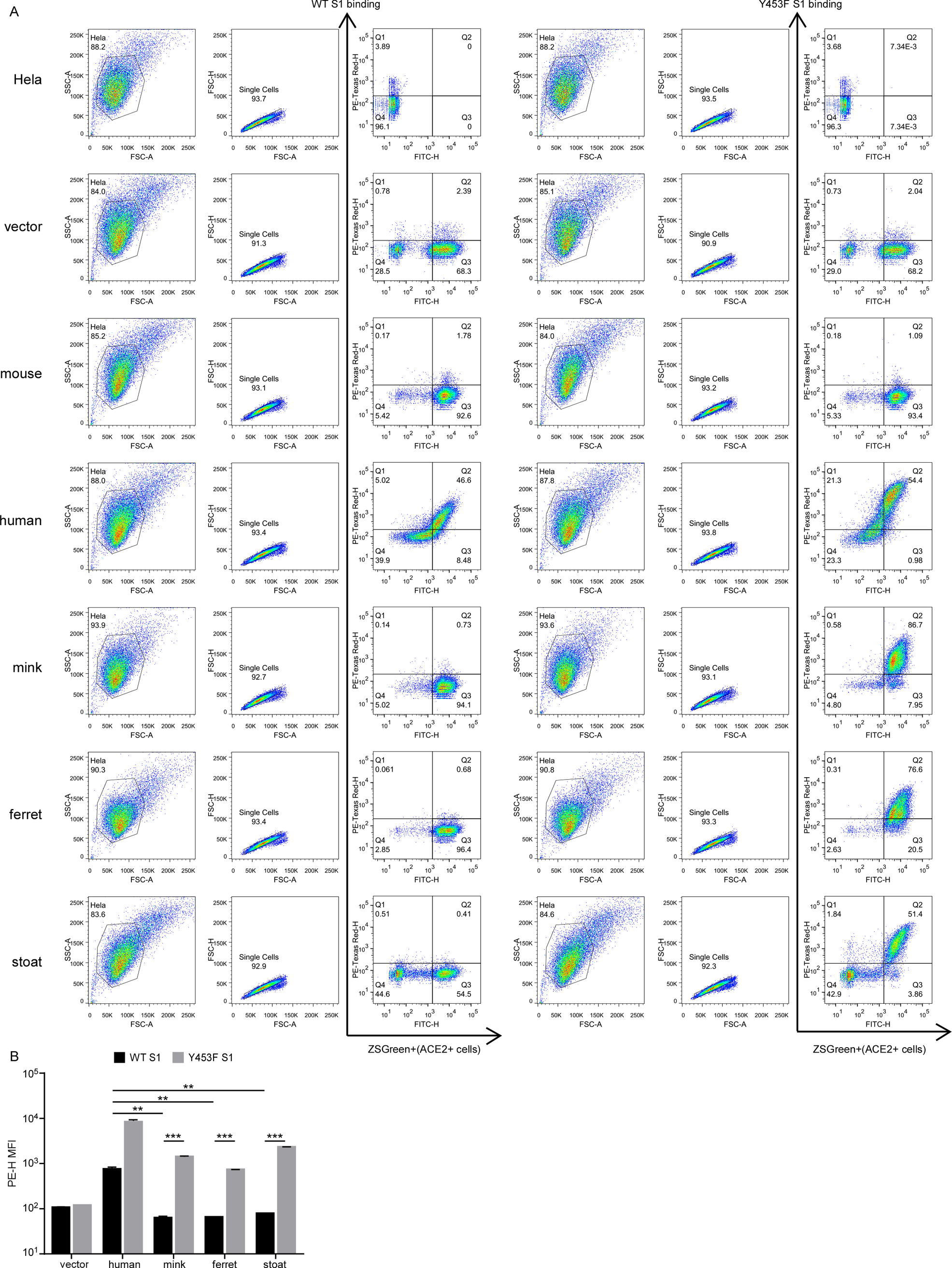

Supplement: S6 Fig — (A) The main cell population was identified and gated on Forward and Side Scatter. Single cells were further gated on FSC-A and FSC-H. The gated cells were plotted by FITC-H (zsGreen, as the ACE2-expressing population) and PE-Texas Red-H (S1-Fc bound population). The PE-Texas Red-H positive cell population was plotted as a histogram to show the S1-Fc positive population as in Fig 2D. The binding efficiency was defined as the percent of S1-Fc binding cells among the zsGreen-positive cells. Shown are FACS plots representative of those used for the calculations of binding efficiencies of ACE2 orthologs with S1-Fc. (B) The MFI values of ACE2+ cells incubated with S1 proteins as indicated were plotted. Shown are FACS plots representative of those used for the calculations of binding efficiencies of ACE2 orthologs with S1-Fc. All binding assays were performed in duplicate. (TIF) [file ppat.1010053.s006.tif]

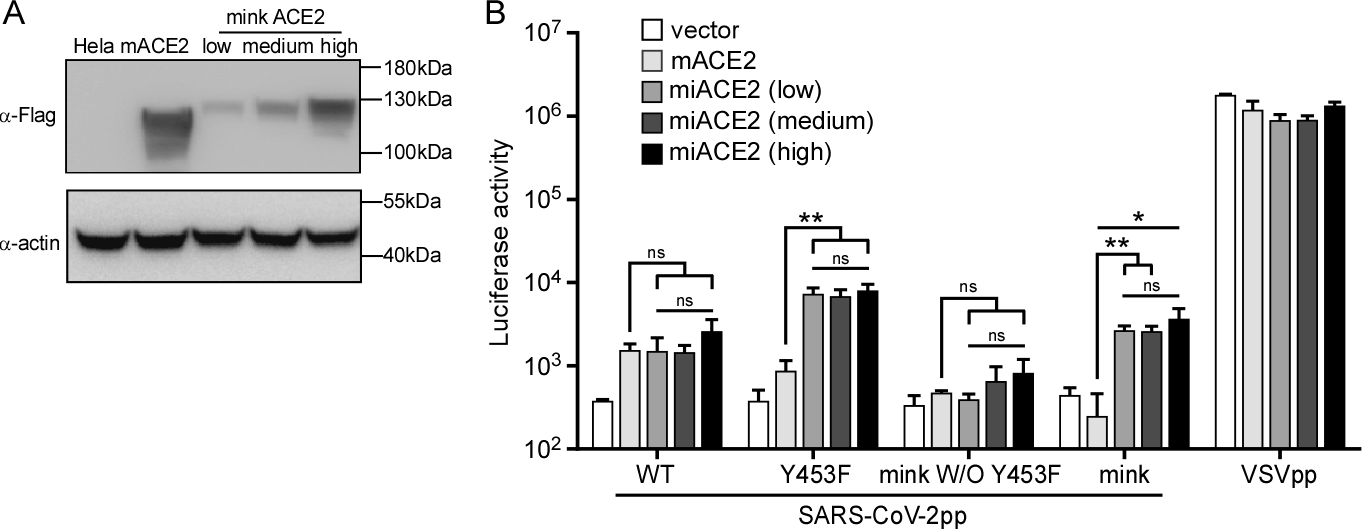

Supplement: S7 Fig — (A) A549-mink ACE2 cells were sorted into three population based on mink ACE2 expression (high, medium and low). Western blotting assay was performed to validate the mink ACE2 expression in these three populations. (B) A549 cells, A549-mACE2 cells, A549-miACE2 cells (high, medium or low) were infected with indicated SARS-CoV-2 pseudoparticles. Two days after infection, cells were lysed and luciferase activity determined. All infections were performed in triplicate, and the data are representative of two independent experiments (mean ± SD). ns, no significance, *, P < 0.05, **, P < 0.01. Significance assessed by one-way ANOVA. (TIF) [file ppat.1010053.s007.tif]

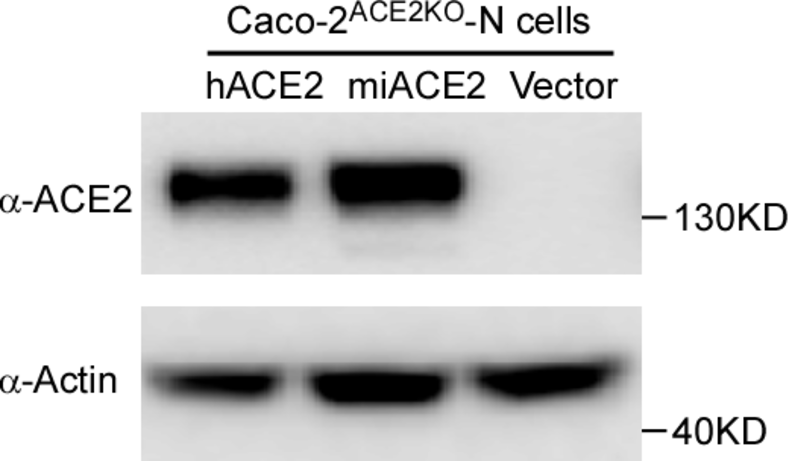

Supplement: S8 Fig — Caco-2ACE2KO-N cells were transduced with lentivirus expressing human ACE2 or mink ACE2. Immunoblotting assay was performed to detect the expression of human ACE2 and mink ACE2. Actin was used as the loading control. These experiments were independently performed twice with similar results. (TIF) [file ppat.1010053.s008.tif]
